# Identifying persons at risk for developing type 2 diabetes in a concentrated population of high risk ethnicities in Canada using a risk assessment questionnaire and point-of-care capillary blood HbA_1c_ measurement

**DOI:** 10.1186/1471-2458-14-929

**Published:** 2014-09-08

**Authors:** Chip P Rowan, Lisa A Miadovnik, Michael C Riddell, Michael A Rotondi, Norman Gledhill, Veronica K Jamnik

**Affiliations:** 358 Norman Bethune College, York University, 4700 Keele St., Toronto, Ontario M3J 1P3 Canada; 347 Norman Bethune College, York University, 4700 Keele St., Toronto, Ontario M3J 1P3 Canada; 364 Norman Bethune College, York University, 4700 Keele St., Toronto, Ontario M3J 1P3 Canada; 356 Norman Bethune College, York University, 4700 Keele St., Toronto, Ontario M3J 1P3 Canada; 355 Norman Bethune College, York University, 4700 Keele St., Toronto, Ontario M3J 1P3 Canada

**Keywords:** (Up to 10) pre diabetes, Screening, Physical activity, Body mass index, Point of care

## Abstract

**Background:**

Amidst the growing health care burden created by diabetes, this study aimed to assess the utility of a prediabetes/type 2 diabetes risk questionnaire in high risk ethnic communities in Toronto Canada.

**Methods:**

Participants (*n* = 691) provided questionnaire responses and capillary blood tests collected via fingerstick and results were analysed for HbA_1c_ using the Bio-Rad in2it point-of-care device. The Bland-Altman method was used to compare point-of-care HbA_1c_ analysis (Bio-Rad, boronate affinity chromatography) to that using high performance liquid chromatography. ANOVA and linear regression were performed to investigate the relationship between questionnaire and blood data.

**Results:**

Mean (±SD) HbA_1c_ was 5.99% ± 0.84 and the Bland-Altman analysis revealed no significant biases HbA_1c_ (bias = 0.039, 95% limits of agreement = -1.14 to 1.22). ANOVA showed that with increasing risk classification based on questionnaire answers (with the exception of "moderate"-to-"high"), there was a significant increase in mean HbA_1c_ (Welch Statistic 30.449, p < 0.001). Linear regression revealed that the number of high risk parents, age category, BMI, physical activity participation and previous diagnosis of high blood sugar were significant contributors (p < 0.05) to the variance in HbA_1c_.

**Conclusions:**

Though not a substitute for established diagnostic protocols, the use of a risk questionnaire can be an accurate, low cost, educational and time efficient method for assessment of type 2 diabetes risk. The early detection of prediabetes and type 2 diabetes is vital to increased awareness and opportunity for intervention with the goal of preventing or delaying the progression of type 2 diabetes and the known associated complications.

**Electronic supplementary material:**

The online version of this article (doi:10.1186/1471-2458-14-929) contains supplementary material, which is available to authorized users.

## Background

Type 2 diabetes mellitus in Canada is rapidly progressing into a dire situation with enormous public health and economic implications. As of 2009, approximately 2.4 million Canadians were living with a diagnosis of type 2 diabetes, a number that is expected to grow to approximately 3.7 million by 2019 [1]. Perhaps of greater concern is that approximately 20% of type 2 diabetes cases remain undiagnosed in addition to more than 5 million Canadian adults with prediabetes [1]. The economic burden of diabetes and its antecedent condition, prediabetes, is unsustainable moving into the future. The Canadian Diabetes Association (CDA) reports that the 2009 cost of type 2 diabetes and its complications was $12.2 billion and forecasts an additional $4.7 billion in costs by 2020 [2]. This projected cost underscores the urgent need to identify those who are undiagnosed or who have prediabetes so that progression toward a type 2 diabetes diagnosis can be avoided or, at the very least, delayed.

Type 2 diabetes is acknowledged to be a preventable condition, a premise that is substantiated by seminal randomized clinical trials [3–5]. The Diabetes Prevention Program is widely recognized as a landmark research study which showed a 58% reduction in diabetes incidence over a 4 year time frame among individuals with prediabetes who participated in a lifestyle intervention involving physical activity and nutritional counselling [3]. From a public health perspective, the first step in the prevention process, should be the identification of frequently occurring risk factors for type 2 diabetes, including: age ≥40 years, family history of type 2 diabetes, history of gestational diabetes, poor blood lipid profile, hypertension, abdominal obesity, physical inactivity and being a member of a high-risk population such as persons of Aboriginal, South Asian, Chinese, or African descent [6]. Of particular interest are those risk factors that are directly modifiable through lifestyle interventions such as abdominal obesity, hypertension, blood lipid profile and physical activity level. Identification of these risk factors not only provides an assessment of diabetes risk, but also acts as an important first step providing awareness and education with the goal of eliciting healthy lifestyle changes. As it pertains to disease management [7, 8], the type and volume of physical activity has been widely studied among those with type 2 diabetes but little is known about the result of physical activity interventions for those with prediabetes. Also, programs that are designed to be culturally specific and community-based may provide a unique opportunity to offer screening and intervention opportunities to individuals at highest risk [9], although the effectiveness of such programs as interventions has yet to be studied. An effective exercise prescription showing an appreciation for the various physiological adaptations to regular aerobic and resistance training as they pertain to type 2 diabetes prevention is essential [10]. For persons with prediabetes and type 2 diabetes, the CDA [6] and American Diabetes Association (ADA) [11] recommends participation in a minimum of 150 minutes of moderate intensity (50-70% age-predicted maximum heart rate) aerobic physical activity per week such as brisk walking, cycling or water aerobics in addition to resistance training exercises 2–3 times per week using weight machines, free weights or body-weight exercises.

There have been several attempts to create a front-line risk assessment tool that can readily identify those at highest risk for developing type 2 diabetes. The Finnish Diabetes Risk Score (FINDRISC) questionnaire was generated in Finland as a product of the Finnish diabetes prevention study and it has been modified for use in several different countries, such as the Canadian Diabetes Risk Questionnaire (CANRISK) questionnaire by the Public Health Agency of Canada. The FINDRISC questionnaire was selected as a template based on its ability to effectively detect impaired glucose metabolism among Scandinavian populations [12, 13]. CANRISK was modified for the Canadian population with the goal of accounting for the greater ethnic diversity compared to that of Finland [14]. CANRISK also includes questions about level of education and, for women, if they had given birth to a large baby (over 9 lb) both of which are known to be associated with type 2 diabetes risk [14]. Neither the FINDRISC, nor the CANRISK questionnaires used HbA_1c_ as the primary assessment tool for glycemic control, although CANRISK did include HbA_1c_ measures in a sub-population [12, 14, 15].

Regardless of the questionnaire being used, a fast, simple and low-cost option for detecting type 2 diabetes risk that is validated against standardized diagnostic blood test scores is an essential tool for programs that aim to reduce the incidence of type 2 diabetes. The purpose of this investigation was to test the hypothesis that a pen and paper risk questionnaire could accurately capture type 2 diabetes risk factor profiles and stratify a person’s overall risk for developing type 2 diabetes that is comparable to results of a capillary blood test for HbA_1c_ collected via fingerstick.

## Methods

### Study design

The Prediabetes Detection and Physical Activity Intervention Delivery (PRE-PAID) project focuses on the detection of individuals at high risk for developing type 2 diabetes using a community-based public health approach. The mandate of the PRE-PAID program was to focus efforts on ethnicities known to be at elevated risk for developing type 2 diabetes, which include persons of South Asian, African-Caribbean, Chinese and Aboriginal descent.

Selected communities had an elevated prevalence of type 2 diabetes and a concentrated population of high risk ethnicities. Demographic information was taken from the Institute for Clinical Evaluative Sciences diabetes atlas for the city of Toronto which provided information about diabetes incidence and prevalence by neighbourhood as well as a breakdown of the population by ethnicity [16]. Study participants were recruited through an established network of community partnerships with various organizations that provide public health-related programs to their constituents. Participants were recruited through printed materials, e-mail distribution lists and public diabetes screening events held in high-traffic areas such as shopping malls and community health centres. All participants provided written, informed consent prior to collection of data and all protocols utilized by the PRE-PAID project were approved by the York University Human Participants Review Committee.

### Questionnaire design

The FINDRISC and CANRISK questionnaires provided a detailed and well-established framework upon which the PRE-PAID risk questionnaire was modeled. Slight alterations from the CANRISK questionnaire were made to minimize participant burden by removing questions about fruit and vegetable consumption, level of education and giving birth to a large baby. The PRE-PAID investigators opted to streamline the time taken to complete the questionnaire due to the fact that the capillary blood testing immediately followed its completion and some participants may have been lost due to the additional 15 minute commitment for the blood testing component. The questionnaire was also modified in order to include more detailed information (frequency and intensity) regarding the physical activity habits of those completing the questionnaire. These changes were also adopted as a result of the published validation of the CANRISK questionnaire which showed that the question regarding fruit and vegetable consumption, physical activity and macrosomia (birth to a large baby) were not significant contributors to their logistic regression model [15]. The PRE-PAID questionnaire is included as Additional file 1. Upon completion of the seven questions, an overall risk score was tabulated, based on a scoring paradigm similar to that of CANRISK, placing individuals into one of five different risk categories; "Small" (score 0–6), "Moderate" (score 7–11), "High" (score 12–14), "Very High" (score 15–20) and "Extreme" (score over 20). Trained members of the research team assisted study participants with questionnaire completion, and all questionnaire responses were based on self-reported information. BMI charts were provided to simplify the estimation of BMI from body mass and height (kg/m^2^). Participants were only required to complete the questions that contributed to the calculated risk score. The PRE-PAID questionnaire included space to self-report specific values for height, body mass, age and waist circumference. The inclusion of these values was encouraged to allow future analysis of participant demographics, but not required to attain a complete risk score.

### Study participants

Persons were considered eligible for inclusion if they were over 18 years of age and if they did not possess any condition that would preclude them from having a capillary blood test to assess their glycemic control. English language proficiency was encouraged but not essential as the questionnaire was translated into Chinese (simplified and traditional), Punjabi, and Hindi. A total of 691 individuals were recruited in this study.

### Blood testing

Point-of-care fingerstick capillary blood testing was performed to validate the risk questionnaire outcomes. HbA_1c_ was selected as the primary blood biomarker because it is a simple, minimally invasive measure that does not require the person to be in a fasted state, thus allowing for flexible testing capabilities. HbA_1c_ is an indicator of three-month glycemic control and is less variable than fasted blood glucose sampling on a day-to-day basis. HbA_1c_ has also been adopted as part of the prediabetes and type 2 diagnostic criteria by CDA as well as the American Diabetes Association (ADA) [6, 17]. For these reasons, HbA_1c_ is a highly appropriate biomarker for the evaluating the validity of the risk questionnaire.

HbA_1c_ was analyzed using the Bio-Rad in2it (Bio-Rad Laboratories, Hercules, CA) point-of-care device and boronate affinity chromatography. All capillary blood samples were collected by a trained phlebotomist and sterile techniques were utilized in accordance with York University biosafety and ethics requirements. In a sub-set of individuals, a second HbA_1c_ sample (from the same fingerstick) was collected using Bio-Rad capillary tubes for analysis using high-performance liquid chromatography (HPLC), a standardized HbA_1c_ analysis criterion method that is in accordance with National Glycohemoglobin Standardization Program regulations. The HPLC analyses described above were performed by Clearstone Central Laboratories (Mississauga, ON) using the Bio-Rad Variant II Hemoglobin testing system.

Results of the HbA_1c_ tests were interpreted based on the 2013 Canadian Diabetes Association clinical practice guidelines diagnostic criteria [6] which define prediabetes using an HbA_1c_ range of 6.0-6.4% and type 2 diabetes using an HbA_1c_ range of ≥6.5% [6]. It should be noted that the ADA use an HbA_1c_ range of 5.7-6.4% for prediabetes and ≥6.5% for diabetes [17]. Participants were informed that the results from the blood tests taken for the PRE-PAID project were not designed to provide medical diagnosis of prediabetes or type 2 diabetes. Individuals who had HbA_1c_ scores ≥6.5% were provided with a letter describing their results and encouraged to see their primary care physician for further confirmatory testing.

### Statistical analyses

**Figure 1:**
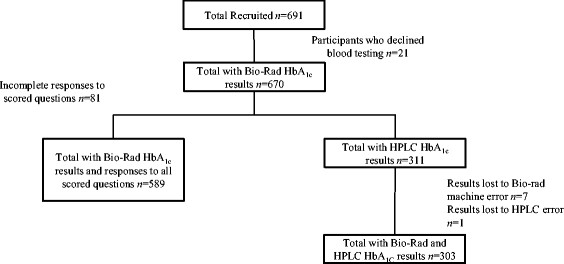
**Participant recruitment and inclusion in the data analyses.**

Descriptive statistics as well as frequencies of questionnaire responses were analyzed for all participants who completed the risk questionnaire. Various exclusions within the dataset took place for further analyses based on missing data that was attributable to participant error, data entry error, or the participant’s unwillingness to provide a blood sample. Figure 1 shows the participant flow diagram for the PRE-PAID risk questionnaire administration.

A comparison of the two methods for determining HbA_1c_ was performed using the Bland-Altman method [18] to detect any potential biases between the two methods of analysis. All analyses described in this investigation were performed using a two-sided 5% level for significance.

ANOVA with post-hoc pairwise comparisons was performed using Tamhane’s T_2_ approach, which allows for unequal variances to compare risk classification based on the questionnaire score to mean HbA_1c_ measured using the Bio-Rad device. Prior to analysis, the "Very High" and "Extreme" groups were merged because of a very small number of participants falling within the "Extreme" classification. From a clinical perspective, individuals within both of the highest risk groups would be strongly encouraged to visit a physician for further assessment regardless. In addition to the ANOVA, additional analyses including the area under the receiver-operator curve and examination of sensitivity and specificity were performed to examine reliability. These analyses used a cut-point of 6.5 which corresponds to the "moderate" risk category to better describe the ability of the risk questionnaire to predict dysglycemia defined by HbA_1c_ ≥ 6.0%.

Finally, step-wise, backward elimination linear regression was performed to quantify the amount of variance in HbA_1c_ values that was attributable to each of the variables included on the risk questionnaire. The Bland Altman plots were performed using GraphPad Prism 6 and all other analyses were performed using SPSS version 20.

## Results

### Study participants

A total of 691 participants completed the risk questionnaire. The participants were primarily female (71%) and 83% of participants reported having two parents from an ethnicity known to be at high-risk for developing type 2 diabetes.

### Questionnaire results

The mean overall risk score for all participants was 9.7 ± 5.3 (mean ± SD) which corresponds to the "Moderate" risk classification. Notable findings include 44.1% of the respondents reported to be physically active 3 or more times per week compared to 33.1% who reported once or twice per week and 22.8% reported being physically active rarely or never. In terms of body composition, self-reported BMI results show that 43.6% fall into the normal range (BMI <25) while 33.3% were overweight (BMI 25–29) and 23.1% were obese (BMI ≥30) based on World Health Organization BMI cut points for adults [19]. The adjusted cut points for Asian populations [20] were not used because of the heterogeneity of the participant population. Also of note, 28.9% of participants reported having been told that they have high blood pressure by a physician and 14.8% of participants responded "yes" to having been told by a physician that they have high blood sugar. Finally, 65.5% of participants noted that they had a family history of diabetes and among these participants, 68.4% noted that this was an immediate relative (mother, father, brother, sister or own child). Based on the overall risk score, 30.2% of participants fell into the "Small" risk category, 33.1% into the "Moderate" risk category, 16.5% into the "High" risk category, 15.4% into the "Very High" risk category, and 4.8% into the "Extreme" risk category. The frequency data from the questionnaire responses are summarized in Table 1 along with descriptive data for questionnaire and blood test outcomes.

**Table 1:** **Summary of questionnaire and capillary blood testing outcomes**

Questionnaire item	Response	Frequency (***n***)	Percent (%)
Sex	Female	418	71
	Male	171	29
Number of high risk parents	None	86	14.6
	One	14	2.4
	Two	489	83
Age categories	<40	190	32.3
	40-44	68	11.5
	45-54	142	24.1
	55-64	131	22.2
	65+	58	9.8
BMI (Kg/m^2^)	<25	257	43.6
	25-29.9	196	33.3
	30+	136	23.1
Waist circumference^a^	Healthy	245	41.6
	Overweight	150	25.5
	Obese	194	32.9
Physical activity participation	3+ Times per week	260	44.1
	1 or 2 Times per week	195	33.1
	Rarely or Never	134	22.8
High blood pressure	No	419	71.1
	Yes	170	28.9
High blood sugar	No	502	85.2
	Yes	87	14.8
Family history of diabetes	None	203	34.5
	2^nd^ degree relative	122	20.7
	1^st^ degree relative	264	44.8
Risk classification	Small	178	30.2
	Moderate	195	33.1
	High	97	16.5
	Very High	91	15.4
	Extreme	28	4.8
Descriptive variables	*n*	Mean	Std. Deviation
Bio-Rad HbA_1c_ %	670	5.99	0.84
HPLC HbA_1c_ %	311	5.81	0.97
Questionnaire score	589	9.7	5.4
^*^Waist circumference range	Males	Females	
Healthy	<94 cm	<80 cm	
Overweight	94-102 cm	80-88 cm	
Obese	>102 cm	>88 cm	

^*^Waist circumference cutoffs.

### Blood results

A total of 670 people went on to provide a capillary blood sample using the Bio-Rad point-of-care device after completing the risk questionnaire. From this group, a subset of 311 provided a sample for analysis using HPLC. The mean Bio-Rad HbA_1c_ (*n* = 670) was 5.99 ± 0.84% while the mean HPLC value was 5.81 ± 0.97%.

**Figure 2:**
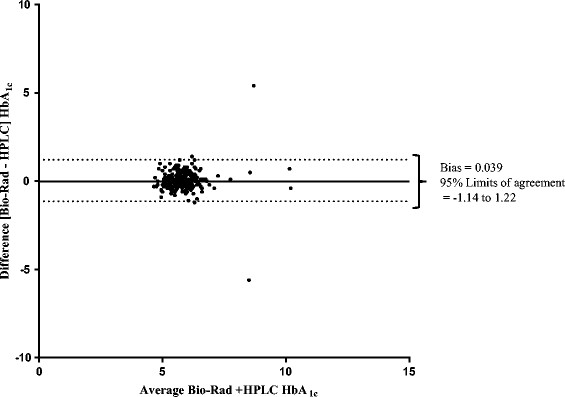
**Bland-Altman plot comparing Bio-Rad and HPLC HbA**_**1C**_ **analyses.**

Analysis comparing the HbA_1c_ scores collected using the two different methods (Bio-Rad and HPLC) took place for 303 persons and Figure 2 provides a Bland-Altman plot that describes the relationship between the two test measures. A non-significant bias of 0.039 (95% limits of agreement = -1.14 to 1.22) was observed when comparing absolute HbA_1c_ scores using both devices (*n* = 303).

### Comparison of risk questionnaire and blood outcomes

**Figure 3:**
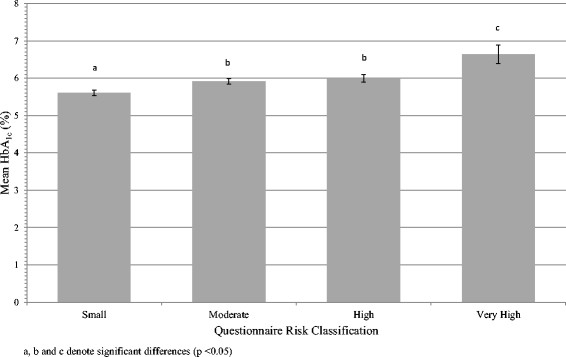
**Risk classification based on questionnaire score compared to mean HbA**_**1c**_ **[(%) ± 95% Confidence Interval] measured using the Bio-Rad in2it device.**

For this portion of the analysis, participants were excluded if they were missing data for any component of the risk score on the questionnaire or if they did not have a Bio-Rad HbA_1c_ value. A total of 589 participants were included in the analysis. A one-way ANOVA was performed to describe the relationship between HbA_1c_ values and overall risk score classification. The results of the ANOVA revealed that the assumption of homogeneity of variance was violated (Levene’s statistic 20.6, p < 0.001). Welch tests were performed which showed that there were significant differences between groups (Welch Statistic 30.449, p < 0.001). Post-hoc comparisons, using Tamhane’s T_2_ approach, which allows for unequal variances, revealed only the "Moderate" and "High" risk groups were not significantly different (p = 0.72) from each other in terms of mean HbA_1c_. The results of the ANOVA are presented in Figure 3.

The results of the step-wise, backward elimination linear regression analysis (n = 589) revealed that the number of high risk parents (standardized β = 0.15, p < 0.001), age category (standardized β = 0.12, p < 0.001), BMI (standardized β = 0.11, p < 0.001), physical activity participation (standardized β = 0.12, p < 0.001) and previous diagnosis of high blood sugar (standardized β = 0.28, p < 0.001) were all significant contributors to the variance in Bio-Rad HbA_1c_. The R^2^ for this model was 0.235. Results from the linear regression are shown in Table 2. The area under the receiver-operator curve (AUC) was 0.716 using the definition of dysglycemia as HbA_1c_ ≥6.0%. The sensitivity and specificity using a score of 6.5 as a cut-point were 0.853 and 0.435, respectively. This shows that, if a person scored 7 or higher (there are no half points allocated) which corresponds to "moderate" risk or higher, then the likelihood of detecting true dysglycemia is promising. These results resemble the values for moderate risk and mirror the incremental reduction in sensitivity with increased cut-point score selected for the sensitivity/specificity analysis observed using the CANRISK questionnaire [15].

**Table 2:** **Results from the full step-wise, backward elimination linear regression model**

Questionnaire item	Standardized beta	t	Sig.
Increasing number of high risk parents	0.15	3.78	<0.001
Increasing age category	0.12	2.87	<0.001
Increasing BMI	0.11	2.42	0.02
Increasing waist circumference	0.06	1.25	0.21
Decreasing physical activity participation	0.12	3.30	<0.001
High blood pressure	0.02	0.53	0.60
High blood sugar	0.28	7.38	<0.001
Family history of diabetes	0.05	1.23	0.22

R^2^ = 0.235, Adjusted R^2^ = .224.

Although participants were made aware that this project was not intended to diagnose prediabetes or diabetes, it was still possible to ascertain valuable information regarding the detection of participants previously unaware (undiagnosed) of their high blood sugar through comparison of their HbA_1c_ value to their response to the question, "have you ever been told by a doctor or nurse that you have high blood sugar?". This process showed that 79.7% of participants with an HbA_1c_ ≥ 5.7% (ADA prediabetes cut point), 75% with an HbA_1c_ ≥ 6.0% (CDA prediabetes cut point) and 61.7% with an HbA_1c_ ≥ 6.5% had never been told that they had high blood sugar.

## Discussion

When comparing the classifications of diabetes risk based on questionnaire overall risk score to HbA_1c_ values, significant and expected increases in HbA_1c_ were observed as participants progressed from a risk classification of "Small" toward "Very High" or "Extreme". After collapsing the "Very High" and "Extreme" groups, the only groups that did not significantly differ were the "Moderate" and "High" risk groups. Of particular interest, those in the "Small" risk category based on the questionnaire responses had average HbA_1c_ values corresponding to the healthy glycemic control while those in the "Moderate" risk group had average HbA_1c_ values that were approaching a state of prediabetes based on the CDA diagnostic criteria [6]. Furthermore, these "Moderate" risk individuals would be in the prediabetes range based on ADA standards which define prediabetes using an HbA_1c_ of 5.7-6.4% [17]. Those in the "High" risk group, based on their questionnaire responses, had corresponding blood test scores with an average HbA_1c_ value at the cusp of the prediabetes classification according to the CDA range (mean HbA_1c_ of "High" risk group = 5.99%, CDA Range = 6.0-6.4%) and in the middle of the ADA prediabetes range (HbA1c of 5.7-6.4%) . Finally, those in the "Very High" risk group had average HbA_1c_ values (Mean HbA_1c_ = 6.6%) in the diabetes range (≥6.5%) based on both the CDA and ADA guidelines. Another related finding, with substantial clinical significance, was the extent to which the screening process identified individuals who were previously unaware of their poor glycemic control. With 75% of persons in the prediabetes range and ~62% of persons in the diabetes range based on their HbA_1c_ report having never been told by a physician or nurse that they had high blood sugar, serious implications regarding the need for diabetes and prediabetes screening are magnified.

Further investigation into the relationship between questionnaire outcomes and blood values using multivariate linear regression revealed that, in descending order of standardized beta values, previous diagnosis of high blood sugar (standardized β = 0.28), number of high risk parents (standardized β = 0.0.15), physical activity participation (standardized β = 0.12), age category (standardized β = 0.12), and BMI (standardized β = 0.11) were all independent significant contributors to the variability in HbA_1c_. While the R^2^ statistic suggests that the model only explains 23.5% of the variance in HbA_1c_, a receiver operator characteristic (ROC) analysis was performed and the area under the curve (AUC) was 0.716 using dysglycemia (HbA_1c_ ≥6.0%) as the primary outcome with the intention of drawing comparisons to existing diabetes risk questionnaires. The observed AUC for the ROC analysis is consistent with findings from the CANRISK (AUROC = 0.75) and FINDRISC (AUROC = 0.648 for men, 0.659 for women) questionnaires for the prediction of dysglycemia (prediabetes + type 2 diabetes) [12, 15]. The relatively low R^2^ of this model identifies a legitimate area of further investigation to decipher what may be contributing to the remainder of the variance in HbA_1c_ within high risk populations. Interestingly, an analysis of the CANRISK questionnaire outcomes found that the response to their physical activity participation question was not a significant contributor to their model [15]. This disparity between the CANRISK questionnaire and the PRE-PAID questionnaire, with respect to the significance of physical activity in the model is likely due to the fact that the PRE-PAID questionnaire had an altered version of the question which was more descriptive in its assessment of physical activity and ascertained information about physical activity frequency. These findings and the corresponding standardized beta values will be used in the future to establish weighted responses on the questionnaire with the goal of enhancing its predictive value.

The utilization of HbA_1c_ as the primary blood biomarker for confirmation of risk provided the investigators with a great deal of freedom in scheduling recruitment and screening events. Through the use of minimally-invasive point-of-care capillary blood testing, a broad pool of potential participants was reached. The ability to test blood in a non-fasted state and provide rapid results made this test more accessible and appealing to potential participants, thus enhancing the efficacy of recruitment efforts. The comparison between the Bio-Rad device and HPLC revealed no significant bias between the two measures which led to the decision to use the Bio-Rad samples (n = 589 with Bio-Rad values versus 304 with HPLC) for the data analysis comparing blood results to questionnaire outcomes via ANOVA and linear regression. Further, the accordance between the two HbA_1c_ supports the use of minimally-invasive point-of-care capillary blood testing for future type 2 diabetes and prediabetes detection initiatives that are focused on screening, awareness and education. These tests may be accessible to a larger population because they can be performed at lower costs and less intrusive to persons at risk while providing relatively accurate information, especially when used in conjunction with a risk questionnaire.

One of the primary limitations of this investigation is the demographics of the sample. In an ideal setting, a more diverse sample would provide an opportunity to enhance the validation of the PRE-PAID questionnaire for use on a broader population. In spite of this, the mandate of the PRE-PAID investigators and the funding agencies was to reach those at highest risk for developing type 2 diabetes, thus leading to more targeted recruitment efforts. The concentrated efforts aimed at reaching high risk ethnicities supports the notion that the PRE-PAID questionnaire provides a unique and appropriate tool for use in public health screening initiatives that target these populations. Another limitation of this investigation is the fact that all responses to the risk questionnaire were self-reported and several studies have shown that individuals tend to under-report their weight and waist circumference [21, 22] while over-reporting their physical activity habits [23, 24]. Although this may be a limitation, it is important to realize that during many public health initiatives, questionnaires are distributed in a similar manner and self-reported data is easier and less expensive to obtain when compared to actual measurement of the various risk factors assessed on the PRE-PAID questionnaire which may require equipment and trained personnel. Another limitation of the investigation pertains to the wording of questions assessing previous diagnoses of high blood pressure, blood sugar and family history of diabetes. Those who "didn’t know" were given a score of zero. Moving forward, a more conservative approach should be taken so that those who do not know how to respond, are assumed to possess that risk factor and therefore receive a score for that question, thus contributing to their overall risk score. Finally, there have been some studies that have documented the presence of hemoglobinopathies or other conditions such as iron deficiency which would make the use of HbA_1c_ inappropriate for the assessment of diabetes status [6, 25]. The prevalence of hemoglobinopathies varies greatly depending on country and race but has been reported as high as 10% in some African populations [25]. During the HPLC assessment of HbA_1c_, no participants were identified as having hemoglobinopathies that would warrant their removal from the comparative analysis. It is possible, however, that some of the study participants who only provided Bio-Rad HbA_1c_ samples possessed some form of hemoglobinopathy. Additionally, there may be other factors such as prescription medication which may contribute to altered HbA_1c_ values [26] and it should be noted that this data was not captured by the risk questionnaire during this study. Adding questions regarding medication use would increase the complexity and duration of completing the questionnaire which would increase subject burden.

While the strength of the CANRISK questionnaire lies in its validation using a large, and representative Canadian sample population, the PRE-PAID risk questionnaire has shown to be an effective alternative tool for use among high risk ethnicities in Canada. As a result of the PRE-PAID investigation, the CANRISK questionnaire may enhance its own predictive value if more detailed questions were included with respect to physical activity participation such as; active transport, sedentary time, physical nature of their occupation, structured exercise, leisure time physical activity plus intensity and frequency of daily activities of living. The analysis of the number of high risk parents is also unique to the PRE-PAID questionnaire which provides important information to enhance the identification of risk based on ethnicity. The ultimate goal of this investigation was to develop an inexpensive front-line questionnaire that could accurately assess a person’s risk for developing diabetes.

## Conclusions

Using a simple screening approach involving risk factor identification and HbA_1c_ point-of-care testing, large and diverse population groups become more accessible and the identification of prediabetes can occur earlier. This early detection provides increased awareness and opportunity to individuals allowing them to make important lifestyle changes as quickly as possible with the goal of preventing, or delaying, the progression towards type 2 diabetes and the known associated complications. The potential reduction in type 2 diabetes incidence and prevalence would likely translate into substantial positive implications regarding health care resource utilization and the current socio-economic burden attributed to diabetes.

## Electronic supplementary material

Additional file 1: **Pre-diabetes/Type 2 diabetes screening tool for PRE-PAID.** (PDF 114 KB)
