# Supplementary material for: Identifying persons at risk for developing type 2 diabetes in a concentrated population of high risk ethnicities in Canada using a risk assessment questionnaire and point-of-care capillary blood HbA1c measurement
Source: BMC Public Health. 2014 Sep 8;14:929. doi: 10.1186/1471-2458-14-929 (PMC4168061; doi:10.1186/1471-2458-14-929)
Supplement: Supplementary file 1 — Additional file 1: Pre-diabetes/Type 2 diabetes screening tool for PRE-PAID. (PDF 114 KB) [file 12889_2014_7047_MOESM1_ESM.pdf]

Pre-diabetes/ Type 2 diabetes screening tool for PRE-PAID

This questionnaire is intended for adults aged 18- 64 years who are members of certain ethnic minorities (**Chinese, South Asian, Hispanic, Aboriginal, African or African-Caribbean**) to help find out if you are at risk of developing type 2 diabetes. Please answer as carefully as you can. We will help you complete this form.
What ethnicity are (or were) your biological (blood) parents?

Mother	Father	
☐	☐	White (Caucasian)
☐	☐	Aboriginal
☐	☐	African, African/Caribbean
☐	☐	South Asian (India, Pakistan, Sri Lanka, Nepal, Bangladesh)
☐	☐	Chinese
☐	☐	Other (please specify__________________)

AS YOU GET OLDER, YOUR RISK FOR DIABETES INCREASES
1) What year were you born? _______________________
Select your age group
☐ 18-39 years (0 points)
☐ 40-44 years (0 points)
☐ 45-54 years (2 points)
☐ 55- 64 years (3 points)

BODY SHAPE AND SIZE CAN AFFECT YOUR RISK OF DIABETES
2a) How much do you weigh (either in pounds or in kilograms)?
I weigh ________ pounds *OR* I weigh __________ kilograms

2b) How tall are you without shoes on?
I am ________ feet and _______ inches tall *OR* I am __________centimeters tall

Use the attached height and weight table to find body mass index (BMI)
☐ unshaded area- BMI less than 25 (0 points)
☐ light shaded area- BMI 25-30 (1 points)
☐ darker shaded area- BMI over 30 (3 points)

3) Waist Circumference
I am going to use a tape measure and place it around your waist at the level of the navel (belly button). I will take a measure after you breathing out (do not hold your breath).
__________________ inches *OR* ________cm

☐ male	☐ female
☐ Less than 94cm or 37 inches (0 pts)	☐ Less than 80cm or 31.5 inches (0 pts)
☐ Between 94-102cm or 37-40inches (3 pts)	☐ Between 80-88cm or 31.5-35inches (3 pts)
☐ Over 102cm or 40 inches (4 pts)	☐ Over 88cm or 35 inches (4 pts)

Research Staff NAME: ____

YOUR LEVEL OF PHYSICAL ACTIVITY AND WHAT YOU EAT CAN AFFECT YOUR RISK OF HAVING DIABETES OR "PRE-DIABETES".
4) Daily Activity
Over a typical seven-day period (one week), how many times do you engage in physical activity that is prolonged and intense enough to feel warm or cause sweating and an increase in you heart rate?
☐ At least three times per week (0 points)
☐ Normally once or twice per week (1 point)
☐ Rarely or never (3 points)

HIGH BLOOD PRESSURE AND HIGH BLOOD SUGAR ARE ASSOCIATED WITH DIABETES
5) High Blood Pressure
Have you ever been told by a doctor or nurse that you have high blood pressure *OR* are you taking any medication (pills) for high blood pressure?
☐ No or don't know (0 points)
☐ Yes (2 points)

6) High Blood Sugar
Have you ever been told by a doctor or nurse that you have high blood sugar (i.e. during a health exam) or that you have diabetes or "pre diabetes"?
☐ No or don't know (0 points)
☐ Yes (5 points)

SOME TYPES OF DIABETES RUN IN FAMILIES
7) Family
Have any members of your family been diagnosed with diabetes? (You can circle the one below that have)
☐ No or don't know (0 points)
☐ Yes: grandparent, aunt, uncle, or first cousin (3 points) (ignore this one if the one below applies)
☐ Yes: parent (mother or father), brother or sister or own child (5 points)
Note: your score cannot be greater than 5 points

Likelihood of developing diabetes in the next 10 years:

< 7	small risk
7-11	moderate risk
12-14	high risk
15-20	very high risk
20-25	extreme risk (may already have diabetes)

***SCORE:**

Thank you for filling this questionnaire about diabetes risk. What is the best way for us to contact you if you are eligible and interested in participating in our research study on physical activity and diabetes prevention?

Name ____
Phone number____________________________________
Email address_____________________________________

Physical Activity
And Chronic Disease Unit
York University

*Source: Adapted from the FINDRISK and CANRISK Questionnaires
